# Amygdala Response to Emotional Stimuli without Awareness: Facts and Interpretations

**DOI:** 10.3389/fpsyg.2016.02029

**Published:** 2017-01-10

**Authors:** Matteo Diano, Alessia Celeghin, Arianna Bagnis, Marco Tamietto

**Affiliations:** ^1^Department of Medical and Clinical Psychology, Center of Research on Psychology in Somatic Diseases (CoRPS), Tilburg University, TilburgNetherlands; ^2^Department of Psychology, University of TorinoTorino, Italy; ^3^Department of Experimental Psychology, University of OxfordOxford, UK

**Keywords:** amygdala, attention, hemispatial neglect, blindsight, fMRI neuroimaging, superior colliculus, pulvinar, conscious perception

## Abstract

Over the past two decades, evidence has accumulated that the human amygdala exerts some of its functions also when the observer is not aware of the content, or even presence, of the triggering emotional stimulus. Nevertheless, there is as of yet no consensus on the limits and conditions that affect the extent of amygdala’s response without focused attention or awareness. Here we review past and recent studies on this subject, examining neuroimaging literature on healthy participants as well as brain-damaged patients, and we comment on their strengths and limits. We propose a theoretical distinction between processes involved in *attentional unawareness*, wherein the stimulus is potentially accessible to enter visual awareness but fails to do so because attention is diverted, and in *sensory unawareness*, wherein the stimulus fails to enter awareness because its normal processing in the visual cortex is suppressed. We argue this distinction, along with data sampling amygdala responses with high temporal resolution, helps to appreciate the multiplicity of functional and anatomical mechanisms centered on the amygdala and supporting its role in non-conscious emotion processing. Separate, but interacting, networks relay visual information to the amygdala exploiting different computational properties of subcortical and cortical routes, thereby supporting amygdala functions at different stages of emotion processing. This view reconciles some apparent contradictions in the literature, as well as seemingly contrasting proposals, such as the dual stage and the dual route model. We conclude that evidence in favor of the amygdala response without awareness is solid, albeit this response originates from different functional mechanisms and is driven by more complex neural networks than commonly assumed. Acknowledging the complexity of such mechanisms can foster new insights on the varieties of amygdala functions without awareness and their impact on human behavior.

## Introduction

The amygdala (Amg) is a composite subcortical structure that comprises more than 12 sub-nuclei having distinctive patterns of input–output connections with the rest of the brain (Whalen and Phelps, 2009; Janak and Tye, 2015). This histological and connectional heterogeneity reflects its multifaceted functions. In fact, the Amg has long been known pivotal to emotion processing, but it also serves as an interface between emotion and cognitive functions, including decision-making, learning and attention (Bzdok et al., 2013). Over the past two decades, evidence has accumulated that Amg exerts some of its functions also when the subject is not aware of the content or even presence of the triggering emotional stimulus (Tamietto and de Gelder, 2010). This review will discuss findings related to Amg functions in the absence of stimulus awareness, its afferent and efferent paths mainly involved in non-conscious processing, and the consequences of such processing along several dimensions, including changes in expressive or instrumental actions, psychophysiological and neuroendocrine alterations, or modulation of motivated behavior.

Before entering into each specific issue, there are several preliminary considerations, both theoretical and methodological, about the relevance of studying Amg contribution to emotion processing without awareness. First, Amg functions and circuitry have been well conserved across evolution and appear early during phylogenetic as well as ontogenetic development. For example, the Amg is present in reptiles, birds and mammals (Janak and Tye, 2015), its neurogenesis in humans and other primates is complete at birth (Nikolic and Kostovic, 1986), and its connections laid down by the 2nd week of age (Amaral and Bennett, 2000). Therefore, studying Amg’s role in emotion perception without stimulus awareness enables us to focus on processes representing ‘primitives’ that evolved before, and likely shaped, more sophisticated functions, such as those involved in sustaining perceptual awareness, core feelings or intentionality. Likewise, these primordial Amg functions have been implicated in the specialization of more recent cortical functions across the primate lineage as well as during development and maturation (Leppanen and Nelson, 2009), including present-day organization of the cortical visual system specialized for face and body processing (Johnson, 2005; Liddell et al., 2005; de Gelder, 2006). Hence, this also provides a valuable testing ground for gaging cross-species continuity of functions and comparison. Secondly, examining stimulus properties and categories that evoke Amg activity without awareness, or that by comparison fail to do so, we may be able to abstract from common taxonomies, such as those distinguishing animate from inanimate objects, faces from bodies and so on, to reveal cross-category commonalities between stimulus types and attributes that could not be anticipated by looking at cortical segregation of stimulus categories (de Gelder and Tamietto, 2011; Van den Stock et al., 2014). Lastly, the Amg clearly rests at the intersection between conscious as well as non-conscious emotional processing (Pessoa and Adolphs, 2010). To the extent that these two different modes of processing incoming sensory information co-exist in the brain, assessing which operations the Amg undertakes without awareness helps to unravel functions that may be overridden, modulated or even actively blocked during conscious perception and cortical top-down regulation. This can add valuable insights on the longstanding debate on whether perception with and without awareness are qualitative or quantitatively different phenomena, whether and how they interact and interfere to shape the ultimately conscious representation of the external world, and which are, if anything, the specific evolutionary benefits that determined conservation of emotion processing without awareness across evolution.

The rest of the paper proceeds as follows. We will first introduce a conceptual and terminological distinction between different types of emotion perception without awareness, as they entail profoundly different mechanisms and are sampled through distinctive experimental designs. Second, we will review neuroimaging evidence demonstrating Amg activity for emotion processing without awareness, how this has been interpreted and current controversies and limitations. Third, we will discuss Amg automaticity as a function of time, and how data acquired with high temporal resolution techniques can elucidate and accommodate apparent inconsistencies originating from functional magnetic resonance imaging (fMRI) results. Fourth, we will consider functional and anatomical evidence about the neural networks that seem crucial to convey sensory information to the Amg in the absence of awareness. Firth, we will concentrate on stimulus categories and properties that can be processed non-consciously by the Amg and, finally, we will summarize the behavioral and psychophysiological consequences of emotion perception without awareness. Throughout the review, we will concentrate on vision because this is the system best known in terms of connections with the Amg in human and non-human primates, and because the large majority of human studies investigating Amg’s role in processing emotions without awareness took advantage of visual stimuli.

## Different Types of Unawareness for Emotions and How they are Studied

A host of techniques and experimental manipulations have been used to render emotional stimuli not consciously visible. For example, during backward masking an emotional stimulus (e.g., a facial expression) is briefly presented and then immediately followed by a masking stimulus (e.g., a neutral or scrambled face). If the stimulus onset asynchrony (SOA) between the first (target) and second (mask) stimulus is sufficiently brief, then the observer cannot consciously report the presence or the emotional content of the first stimulus (Esteves et al., 1994; Whalen et al., 1998). Binocular rivalry or continuous flash suppression exploit the mutual inhibition between monocular channels in the primary visual cortex (V1) by presenting different images to the corresponding regions of the two eyes (Pasley et al., 2004; Tong et al., 2006; Yoon et al., 2009). In such conditions, the images alternate in dominating perception and, at any moment, only the dominant image enters awareness, whereas the other non-dominant image goes undetected. Other popular paradigms include dual-task designs where the subject’s attention is engaged in an attention-absorbing task, such as matching judgments between neutral stimuli, while an emotional stimulus is presented at task-irrelevant and unattended locations (Vuilleumier et al., 2001a; Pessoa et al., 2002). In the attentional blink, a rapid stream of stimuli is presented and the subject is required to report the presence of a target stimulus. However, if a second target appears in rapid succession after a first successfully detected target (typically within 500 ms), this latter fails to be reported (Anderson, 2005). Many other paradigms such as priming, Stroop-task, dot-probe designs or the redundant target paradigm have been variably used to sample perception without awareness of emotional and non-emotional stimuli, each with their own advantages and limitations (Mogg et al., 1994; Algom et al., 2004; Pourtois et al., 2006; Beall and Herbert, 2008; Hart et al., 2010; Celeghin et al., 2015c).

Although detailed coverage of these different methods goes beyond the purposes of this review, they can be conveniently grouped in two broad categories that entail different functional mechanisms. Dual-task, attentional blink, visual search or Stroop paradigms render the emotional stimulus not consciously visible by interfering with attentional mechanisms. Psychophysical evidence indicates indeed that visual stimuli outside the focus of attention are not, or are only partially, seen consciously (Mack and Rock, 1998). Accordingly, when attentional resources are engaged in a task, cortical activity that is evoked in visual areas by unattended (i.e., task-irrelevant) stimuli is suppressed or significantly reduced by top-down influences from fronto-parietal regions that control voluntary attention (Beck et al., 2001). We refer to these phenomena as *attentional unawareness*. The processing of emotional information, however, seems less dependent on attentional resources than neutral information (Vuilleumier, 2005). As we will discuss later, this mechanism seems to depend on Amg responsivity.

In contrast, failure to become aware of a stimulus may uniquely depend on sensory reasons, despite attentional selection mechanisms operate normally (Kentridge et al., 2004). For instance, if the energy of the stimulus is below the detection threshold or the exposure time is too brief (subliminal), the stimulus can fail to generate a consciously reportable sensation notwithstanding we attend to it (Savazzi and Marzi, 2002; Dehaene et al., 2006). Backward masking, binocular rivalry or flash suppression do not modulate attention, but interfere temporarily with normal functioning in the ventral occipito-temporal cortex, which is known to be crucial for visual awareness (Macknik and Livingstone, 1998; Williams and Mattingley, 2004; Tong et al., 2006). In this latter case we refer to this type of non-conscious processing as *sensory unawareness*.

Attentional and sensory unawareness are thus qualitatively different phenomena that can be investigated to sample different Amg functions, while still remaining within the domain of non-conscious processes. For example, attentional unawares is well-suited to examine the role of Amg in biasing orientation toward affective stimuli, and to investigate which mechanisms enable Amg to eventually promote privileged access of emotional signals to awareness. Sensory unawareness can instead reveal alternative visual pathway by which the stimuli can reach the Amg, or their impact toward on-going activities, behaviors or judgments, while still remaining unseen. Lastly, patients with brain damage can be an invaluable additional source of information to broaden our wisdom on Amg functions without awareness. Patients with hemispatial neglect due to right temporo-parietal lesions typically fail to pay attention to the contralesional (left) space, and stimuli appearing on that side often go undetected (Driver and Mattingley, 1998). Therefore, the study of Amg response to emotional stimuli in these patients can add insights into the mechanisms governing attentional unawareness. On the other end, patients with cortical blindness ensuing from damage to, or denervation of, the primary visual cortex (V1) offer a case study to investigate the differences between conscious and non-conscious emotion processing due to sensory, as opposed to attentional, causes and the role of Amg therein (Celeghin et al., 2015b). In fact, the V1 lesion in such patients determines permanent blindness to stimuli projected within the scotoma (the visual field region affected by the cortical lesion), also if the stimuli are supra threshold and long-lasting (Celeghin et al., 2015a,c; Weiskrantz, 2009). Lastly, patients with focal damage to the Amg offer the ultimate ground-truth to translate correlational evidence on Amg functions to causation, by observing whether and how the influence of emotional stimuli during attentional or sensory unawareness, as typically reported in healthy subjects during fMRI experiments, is modified or abolished following Amg lesion (Anderson and Phelps, 2001).

## Amygdala Response During Sensory and Attentional Unawareness: Evidence and Limits

Studies reporting Amg response under conditions of attentional or sensory unawareness are summarized in Supplementary Table 1, with indications on the paradigms, stimuli and main findings. Neuroimaging investigations on healthy participants in whom attention was manipulated have often reported that stimulus-evoked activity in the Amg, along with that of other cortical and subcortical structures, is not suppressed when emotional stimuli are unattended (Vuilleumier et al., 2001a; Anderson et al., 2003; Bishop et al., 2004; Williams et al., 2005). Although this has been sometimes interpreted as evidence of strict automaticity in Amg response to emotion, the current evidence is mixed on this issue (Pessoa, 2005; Pessoa et al., 2005; Silvert et al., 2007). For example, Vuilleumier et al. (2001a), showed that Amg activation in response to fearful facial expressions is independent of attention, whereas Pessoa et al. (2002) reported that when attention is engaged elsewhere by a demanding task, Amg response is suppressed. These apparently contradictory results may be partly explained by differences in the tasks and experimental design, which prevent simple or straightforward comparisons. In fact, in the original study by Pessoa et al. (2002), the subjects had to evaluate the gender during trials in which attention was focused on the faces, whereas they had to evaluate the same/different orientation of peripheral bars when faces were unattended. In addition to the focus of attention on faces vs. bars, therefore, also the cognitive load, type of judgment and task requirements varied between the two conditions, whereas in the study by Vuilleumier et al. (2001a) only the focus of attention changed. Also, Pessoa et al. (2002), used a block design, which samples Amg activity across various consecutive repetitions of the same condition and is thus more liable to habituation and less sensitive to physiological responses induced by single events, whereas Vuilleumier et al. (2001a) used an event-related design where the stimuli presented at attended and unattended locations varied randomly between single trials. Another major confounding factor concerns the different responses the Amg displays to various emotion categories. For instance, Williams et al. (2005) found that Amg activity in response to happy facial expressions was greater when faces were attended, whereas for fearful expressions activity was greater when the faces were unattended. Findings gleaned in neuroimaging studies on patients with hemispatial neglect seem more convergent toward Amg ‘automaticity’. Indeed, undetected emotional stimuli on patients’ left side can activate the Amg as well as cortical areas directly connected to it, such as the orbitofrontal cortex or the insula (Vuilleumier et al., 2002; de Gelder et al., 2012; Tamietto et al., 2015). The advantage of addressing the issue of Amg automaticity in neglect patients consists in the fact that no explicit or intentional manipulation of attention is required from the subject, thereby discounting issues related to task differences and attentional load between conditions.

Investigations on sensory unawareness have shown consistently that unseen emotional stimuli elicit activity in the Amg, often along with activity in the superior colliculus and pulvinar (Morris et al., 1998, 1999; Whalen et al., 1998; Critchley et al., 2002; Killgore and Yurgelun-Todd, 2004; Pasley et al., 2004; Williams et al., 2004b, 2006a,b; Liddell et al., 2005; Carlson et al., 2009; Yoon et al., 2009; Juruena et al., 2010; Troiani and Schultz, 2013). But how robust is the Amg response to unseen with respect to seen stimuli? The answer varies markedly amid studies, despite this is an important question to characterize the relative role of Amg during non-conscious processing of emotions. Some reports found indeed that Amg activity during unawareness vs. awareness is the same, others described that in several cases unseen emotions yielded enhanced responses as compared to consciously perceived stimuli (Anderson et al., 2003; Williams et al., 2004b), whereas still others reported greater activity in Amg when participants were aware of emotional expressions (Williams et al., 2006a,b; Amting et al., 2010). Also in this case, methodological differences seem at least partly responsible for the inconsistencies. In fact, assessing the neural bases of emotion perception during sensory unawareness should be based on direct comparisons between consciously and unconsciously, albeit physically identical, stimuli. This type of evidence, however, is difficult to obtain in healthy observers because many paradigms used to make a stimulus invisible for the subject also introduce a spatial and temporal difference from its consciously visible counterpart. At present, investigations on patients with cortical blindness after V1 lesion possibly provide the best opportunity to characterize non-conscious perception of emotions and its neural correlates. These patients can discriminate the emotional content of stimuli that they do not seen consciously, for example guessing whether the stimulus conveys a fearful or happy expression (de Gelder et al., 1999) – a phenomenon known as affective blindsight – and their proficiency is associated with activity in the Amg (de Gelder et al., 1999, 2001; Morris et al., 2001; Hamm et al., 2003; Pegna et al., 2005; de Gelder and Hadjikhani, 2006; Tamietto et al., 2009; Van den Stock et al., 2011b). As it often happens when mixed results are reported, interpretations and theoretical views on the role of Amg tended to cluster along two extremes: those endorsing a strict notion on Amg automaticity and independency from awareness, and those purporting that awareness is a necessary condition for Amg response to occur. Others and we have proposed that neural networks for conscious and non-conscious emotion processing are not entirely segregated (Vuilleumier, 2005; Pessoa et al., 2006; Duncan and Barrett, 2007; Tamietto and de Gelder, 2010; Pourtois et al., 2013). In this context, Amg not only contributes to both modes of processing, but its initial response without awareness actually helps to determine whether the stimulus will reach awareness and how it will modulate behavioral and bodily reactions. Therefore, the temporal dimension of Amg response becomes critical to interpret its role in emotion perception with vs. without awareness, while also offering an additional framework to understand more coherently the seemingly piecemeal findings summarized above.

## Timing of Amg Response: Fast Signals for Slow Measures?

The speed of processing has always been regarded as one hallmark of non-conscious emotion perception (LeDoux, 1996). However, human studies on Amg engagement in emotion processing without awareness typically used fMRI, which has high spatial but poor temporal resolution. In fact, fMRI studies usually average together events occurring during a temporal window of about 2 s, due to the sluggishness of blood oxygen level-dependent (BOLD) response. On the other hand, non-invasive methods with higher temporal resolution in the order of milliseconds, such as EEG and MEG, have traditionally had limitations in sampling neural activity in deep structures like the Amg (Costa et al., 2014). Nevertheless, recent technical advancements in sources analysis, such as the synthetic aperture magnetometry (SAM) and sliding windows analysis increased precision and sensitivity in detecting MEG or EEG signal from deep brain structures.

One early study combining MEG and MRI methods reported early event-related synchronization in the Amg at 20–30 ms after stimulus onset, whereas synchronization in the striate cortex occurred later, at about 40–50 ms after stimulus onset (Luo et al., 2007). A more recent MEG study revealed dissociation between rapid Amg responses to automatic fearful face processing and later responses that interacted with voluntary attention. On each trial, participants had to discriminate the orientation of peripheral bars while task-irrelevant neutral or fearful faces were presented centrally. Rapid enhancement of neural activity in the gamma band triggered by threatening faces (30–60 ms) was independent of task load and occurred under attentional unawareness, whereas emotion processing and attention interacted at later latencies (280–340 ms), subsequent to fronto-parietal activity (Luo et al., 2010). Coherently, two other MEG studies applying dynamic causal modelling (DCM) tested the explanatory power of the automatic Amg response allegedly mediated via subcortical route, versus a model predicting only cortical mediation associated with stimulus awareness over Amg response. A model considering also automatic Amg responses mediated by a subcortical route explained early brain activity better than the model including only cortical access to the Amg, whereas both models had comparable explanatory power at longer latencies (Garrido et al., 2012; Garvert et al., 2014). Therefore, MEG data offer new clues to resolve the longstanding controversy concerning automaticity of Amg response based on fMRI results, as described above (Brosch and Wieser, 2011). On such bases, it seems that Amg automaticity is a function of time, and these findings have been interpreted according to a two-stage model of emotion-attention interaction. Early Amg responses afford initial discrimination between threat and neutral stimuli. These responses occur independently of awareness and attention, possibly because the influence of fronto-parietal cortex in reducing the representation strength of task-irrelevant and unattended emotional information during attentional competition requires more time to be effective. Conversely, later Amg responses are modulated by attention because the same top-down fronto-parietal mechanisms have had sufficient time to enhance the representation of task-relevant and attended information in visual areas. Notably, both the early automatic and later attention-modulated Amg responses lie within the time window of one volume acquisition of fMRI studies, likely resulting in the contamination of the rapid effects. In keeping with such view, EEG recordings have revealed that Amg damage influences emotion perception at two distinct time-windows, one early processing within the P1 time-range, around 100–150 ms post-stimulus onset, and one later component, around 500–600 ms (Rothstein et al., 2010). These findings are consistent with the contribution of Amg in emotion perception at multiple processing stages, and the correlation between the degree of Amg damage and the magnitude of EEG effects at both time-windows supports its causal role.

Admittedly, intracranial electrophysiological recordings offer the most reliable source of evidence concerning both automaticity and temporal properties of Amg response. Three studies addressed this issue by recoding signals directly from electrodes implanted in the Amg of patients undergoing pre-surgical assessment. Pourtois et al. (2010b) employed the same dual-task paradigm previously used by Vuilleumier et al. (2001b) to gage Amg automaticity with fMRI measures. Recordings from lateral Amg showed an early neural response that differentiated between fearful and neutral faces in the 140–290 ms time-range, which occurred independently of, and prior to, attentional effect starting at 700 ms post-stimulus onset. Likewise, Sato et al. (2011) showed greater gamma-band activity in response to fear compared to neutral faces between 50 and 150 ms. Even though this study confirmed early responses to emotional stimuli, sensory or attentional unawareness was not manipulated and stimuli were projected centrally for 1 s. Similarly, Hesse et al. (2016) recorded local field potential in three patients with depth electrodes placed in the Amg and found that early activity in Amg (80–200 ms), but not in other temporal, parietal, or frontal sites, predicts rapid encoding of intentional harm from visual scenes (Hesse et al., 2016). Lastly, a recent study by Mendez-Bertolo et al. (2016) found fast Amg responses beginning 74 ms post-stimulus onset specific for fearful compared to neutral or happy facial expressions. Moreover, fast Amg responses were selective to low spatial frequencies components of fearful faces. This sensitivity to low spatial frequencies is important because it is in keeping with the properties of the magnocellular pathway, which is supposed to relay visual signal to the Amg via a subcortical pathway devoted to fast and non-conscious emotion perception (Vuilleumier et al., 2003a).

The present findings raise two interrelated issues of the utmost relevance. The first concerns how visual information exploitable for non-conscious emotion perception reaches the Amg. The second relates to the encoding properties of the pathway(s) that channel visual information to the Amg without awareness, thereby defining which visual properties, stimulus attributes and categories can undergo non-conscious emotion processing and trigger appropriate responses. In the next two sections we will deal separately with each of these issues.

## Pathways to the Amg Relevant for Non-Conscious Emotion Perception

The canonical pathway for the transmission of visual information from the retina to the Amg passes through the occipito-temporal cortex along the ventral stream, with the main projection originating from the anterior part of the inferior temporal cortex (TE) (e.g., Kravitz et al., 2013). However, prior studies in rodents documented the role of midbrain structures for rapid but coarse processing of affectively laden auditory and visual stimuli, thereby documenting a subcortical pathway to the Amg that bypasses the primary sensory cortices (Jones and Burton, 1976; Campeau and Davis, 1995; LeDoux, 1996; Doron and Ledoux, 1999; Linke et al., 1999; Shi and Davis, 2001). Neuroimaging data on healthy subjects in whom sensory unawareness for emotional stimuli had been induced by experimental manipulations have shown that the superior colliculus, pulvinar, and Amg are commonly activated in response to non-consciously processed emotional signals (Whalen et al., 1998; Morris et al., 1999; Vuilleumier et al., 2003b; Whalen et al., 2004; Liddell et al., 2005; Williams et al., 2006b). Conversely, the primary cortical route that relays visual input to the Amg does not seem to respond significantly during sensory unawareness, but does so when the emotional stimuli are perceived consciously (Pasley et al., 2004; Williams et al., 2006a,b). Unseen facial and bodily expressions have yielded similar findings when presented in the blind fields of patients with affective blindsight. This indicates that a functional subcortical route to the Amg is invovled in emotion perception during sensory unawareness (Morris et al., 2001; de Gelder et al., 2005, 2011; Pegna et al., 2005; Tamietto and de Gelder, 2010; Van den Stock et al., 2011a,b, 2013, 2015a; Georgy et al., 2016). The involvement of the superior colliculus and pulvinar is in keeping with their connectional pattern and physiological properties. Notably, the superficial layers of the SC receive direct retinal input only from the Magnocellular and Koniocellular channels originating from the parasol and bistratified retinal ganglion cells, respectively (Goldberg and Robinson, 1978; Casagrande, 1994; Waleszczyk et al., 2004). Also the medial subdivision of the inferior pulvinar receives direct projections from the retina, in addition to input originating from the superior colliculus and targeting the centro-medial and posterior subdivisions of the inferior pulvinar. Hence, these subcortical structures are ideally positioned to convey visual input to the Amg and bypass transient or permanent inactivation of the visual cortices. Single cell recordings in monkeys provided independent support for the role of the superior colliculus and pulvinar in encoding emotional expressions (Nguyen et al., 2014). Indeed, a subpopulation of neurons in the superior colliculus responds to faces or face-like images also when the images were filtered in low spatial frequency. Moreover, the magnitude and latency of such responses in the superior colliculus to face images correlated significantly with those recorded in the pulvinar. Noteworthy, neurons in the monkey pulvinar respond differentially to specific emotional expressions, as shown in another cell recording study from the same group (Maior et al., 2010).

Granted the role of a subcortical functional pathway to the Amg devoted to processing emotion under sensory unawareness, are these structures also anatomically connected, besides the functional interactions described above? The presence of anatomical connections between the superior colliculus, pulvinar, and Amg has been documented by tracer studies in birds and rodents. Yet similar evidence in primates was lacking until recently (Pessoa, 2005; Pessoa and Adolphs, 2010). Day-Brown et al. (2010) have provided such evidence in the tree shrew, a species considered a prototypical primate, by showing that the dorsal pulvinar, which receives both topographic and diffuse projections from the superior colliculus, also projects to the Amg, thereby forming a disynaptic pathway. The authors proposed that the role of this pathway is to convey non-topographic visual information from the SC to the Amg, with the purpose of ‘alerting the animal to potentially dangerous visual signals’ (Day-Brown et al., 2010). In an attempt to verify whether such anatomical connections also exist in the human brain, we used diffusion tensor imaging (DTI) and tractography techniques to characterize *in vivo* the connectivity between the superior colliculus, pulvinar, and Amg in normal observers and its changes in blindsight patient GY (Tamietto et al., 2012). We found fiber connections between pulvinar and Amg and also between superior colliculus and Amg via the inferior-lateral pulvinar in the healthy observer as well as in patient GY. Unilateral V1 lesion increased fiber connections along this pathway, but only in the patient’s damaged hemisphere, thus providing additional support of the functional role of this subcortical route in conveying visual information critical for affective blindsight and non-conscious emotion perception. A recent tractography study by Rafal et al. (2015) also traced connections between colliculus, pulvinar, and Amg in eight monkeys and twenty healthy human participants. Results in human participates were highly coherent with our prior results, while the authors also reported for the first time anatomical evidence of direct and closely similar connections in the monkey brain.

**FIGURE 1:**
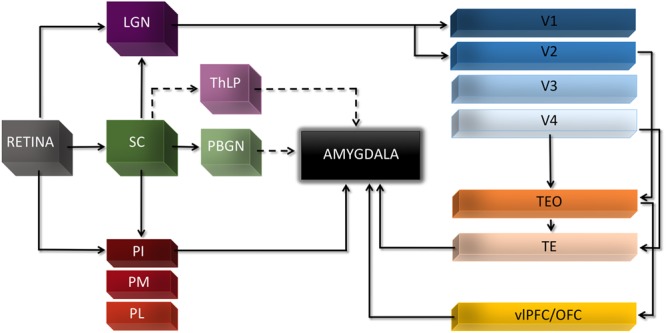
**Major cortical and subcortical visual connections to the Amg.** Dashed lines indicate pathways recently reported in mice and not yet confirmed in human and non-human primates. LGN, lateral geniculate nucleus; OFC, orbitofrontal cortex; PBGN, parabigeminal nucleus; PI, pulvinar inferior; PL, pulvinar lateral; PM, pulvinar medial; SC, superior colliculus; TE, temporal inferior rostral; TEO, temporal inferior posterior; ThLP, thalamus lateral posterior; vlPFC, ventro-lateral prefrontal cortex.

Admittedly, the existence of such subcortical route to the Amg does not exclude other theoretical possibilities or alternative pathways, nor the contribution of cortical areas in different instances of conscious or non-conscious emotion processing (Pessoa and Adolphs, 2010). For example, both the lateral geniculate nucleus and the pulvinar send collateral projections that bypass V1 and target extrastriate visual areas, including areas along the ventral cortical stream that can then relay visual information back to the Amg (Tamietto and Morrone, 2016). Also, two other disynaptic subcortical pathways to the Amg have been recently demonstrated in mice, along with their functional role in triggering innate defensive responses to threatening visual stimuli. Both these pathways originate from the superior colliculus, but one includes the parabigeminal nucleus as intermediate station leading to the Amg (Shang et al., 2015), whereas the other involves the lateral posterior nucleus of the thalamus (Wei et al., 2015). It remains to be established whether these and other potential pathways beyond the well-documented colliculus-pulvinar-Amg play a crucial role for emotion perception without awareness in humans (**Figure 1**).

These two-routes perspectives involving cortical vs. subcortical input to the Amg have been often conceived or presented as alternative to the two-stages account discussed above and emerging from attentional unawareness or analyses of the temporal profile of Amg responses. However, there is no logical contradiction between these two views nor they must be seen as mutually exclusive. Conversely, empirical evidence seems to indicate they co-exist in the intact brain, and they gain new coherence when considered under the light of the distinction between sensory and attentional unawareness introduced above. In fact, when V1 is not able to process visual information normally, because of either experimental manipulation inducing sensory unawareness or permanent damage, the subcortical route seems the primary non-canonical pathway to convey rapidly visual information to Amg and sustain non-conscious emotion processing. During attentional unawareness in healthy subjects or in patients with neglect, however, the visual cortex is normally functioning and coarse magnocellular input can also reach the Amg from cortical areas in the ventral stream through an initial forward sweep (Vuilleumier, 2005; Pourtois et al., 2013). This can afford rapid processing of unattended stimuli prior to voluntary attentional control (Pourtois et al., 2010a,b) or fine-grained and conscious stimulus perception.

## Stimulus Categories and Properties Triggering Amygdala Response Without Awareness

Faces are a privileged *medium* to express emotions during social and non-social interaction. It is therefore not surprising that the wide majority of studies examining emotion perception in human used facial expressions as visual stimuli (e.g., Adolphs, 2002; D’Agata et al., 2011). Likewise, also research on emotion perception without awareness primarily used facial expressions (Morris et al., 1998, 1999; Whalen et al., 1998; Axelrod et al., 2015). This has contributed to the prevailing view that Amg activity during non-conscious emotion perception is selective for facial expressions (Johnson, 2005; de Gelder et al., 2006). However, recent investigation seems to challenge this view from two parallel lines of findings. On the one hand, Amg activity contingent upon sensory and attentional awareness in healthy as well as brain damaged patients has emerged from the use of non-facial stimuli, thereby extending evidence of non-conscious emotion processing to other stimulus categories. Bodily expressions of emotions, both static and dynamic, have been the most extensively studied non-facial stimuli (de Gelder and Hadjikhani, 2006; de Gelder et al., 2006, 2010; Tamietto et al., 2009; Van den Stock et al., 2011a,b, 2013, 2015a,b; Tamietto et al., 2015). Visual stimuli associated to danger in our evolutionary past, such as snakes and spiders, have also been studied during attentional and sensory unawareness. Non-conscious exposure to these stimuli evokes physiological arousal and amygdala response (Carlsson et al., 2004; Wendt et al., 2008; Alpers et al., 2009; Almeida et al., 2015), particularly if participants were phobic to these classes of stimuli, and activated Amg also when unattended because presented in the affected side of patients with hemispatial neglect (Tamietto et al., 2015). On the other hand, the alleged special status of faces in triggering non-conscious perception and Amg activity is at odd with negative evidence when non-emotional facial characteristics, such as gender or identity, are tested during unawareness (Rossion et al., 2000; Negro et al., 2015). Moreover, facial expressions that communicate more complex emotions like guilt or arrogance, whose meaning lays in the socialization process and is less biologically rooted, also fail to undergo non-conscious emotion processing in patients with affective blindsight (Celeghin et al. (2016).

A certain degree of functional similarity between these different stimulus categories, owing to their common suitability in undergoing non-conscious emotion processing and in triggering Amg response, challenges theories exclusively focused on the specific visual features or on the unique role of faces in conveying emotions. In fact, it suggests an approach that cuts across gross physical differences between stimuli, as they exist between facial and bodily expressions, or between these latter and snakes, to concentrate more on common functional properties of these different stimulus classes. The findings reported above thus converge with the idea that non-conscious emotion processing is not specific for faces, but rather for biologically primitive emotional signals that can be encoded from low spatial frequencies, that are clearly associated with action tendencies, and to which we are evolutionary prepared to respond (Tamietto and de Gelder, 2010). Accordingly, complex affective scenes, as derived from the International Affective Picture System (IAPS), cannot be processed non-consciously in patients with affective blindsight (de Gelder et al., 2002) and do not activate Amg under attentional unawareness tested in patients with neglect (Grabowska et al., 2011).

Evidence therefore indicates that processing the emotional value of complex scenes, facial expressions of social emotions, or personal identity from faces depends critically on conscious visual perception and on the detailed processing of the high spatial frequency information that is characteristically performed by the cortical visual system in the ventral stream. We have already discussed findings about fast Amg responses for low but not high spatial frequency fearful expressions (Vuilleumier et al., 2003a; Mendez-Bertolo et al., 2016). In an attempt to determine the causal role and behavioral consequences of Amg activity during non-conscious perception of low spatial frequencies expressions, we have recently tested two patients with affective blindsight in a combined behavioral/fMRI experiment (de Gelder and Tamietto, in press). Neutral and fearful facial expressions were filtered in high or low spatial frequency. We reasoned that, if non-conscious emotion perception during sensory unawareness relies on subcortical pathway to Amg and magnocellular channels, then the patients should display affective blindsight only in response to low spatial frequency images and this above-chance guessing behavior should be associated with Amg activity. Conversely, above-chance guessing should be abolished by high spatial frequency images and Amg response should drop significantly. Preliminary evidence is in keeping with our hypothesis and lends support to the causal role of subcortical structures in affective blindsight and non-conscious emotion perception (de Gelder and Tamietto, in press).

## Consequences of Amg Activity During Non-Conscious Emotion Perception

What are the consequences of Amg activity without stimulus awareness? Do they alter on-going behavior, psychophysiological reactions, or expressive responses toward normally seen environmental stimuli? And, lastly, are these responses felt consciously, even though they cannot be linked to the external triggering event?

Non-conscious perception of emotional stimuli associated with Amg activity often induces behavioral consequences that are associated with characteristic psychophysiological correlates or changes in the bodily state of the unaware observer. These behavioral and psychophysiological outcomes are quantitatively and qualitatively different from those occurring during conscious emotion perception, as they tend to be stronger and faster when awareness is lacking (Williams et al., 2004a; Tamietto et al., 2009, 2015). This suggests that non-conscious perception of emotional stimuli is not simply a degraded version of conscious perception, but a different mode of processing the same stimuli.

For example, emotional stimuli that are unattended, nevertheless interfere with on-going tasks (Eastwood et al., 2003; Hart et al., 2010), and behavioral consequences include delayed disengagement of attention (Georgiou et al., 2005), faster and easier detection than what reported for neutral stimuli, as shown in visual search (Hansen and Hansen, 1988; Ohman et al., 2001), attentional blink paradigms (Anderson, 2005) or in patients with neglect (Vuilleumier and Schwartz, 2001a,b; Williams and Mattingley, 2004; Tamietto et al., 2005, 2007). Notably, damage to the Amg abolishes some of these behavioral effects (Anderson and Phelps, 2001). Similarly, if a neutral stimulus is paired with, or primed by, a non-consciously perceived emotional stimulus, then preferences or attitudes toward the former are shifted accordingly (Niedenthal, 1990; Anders et al., 2009). For instance, consumption behaviors or preference judgments can be influenced by preceding masked facial expressions, despite subjective feelings remain unaltered (Winkielman and Berridge, 2004; Winkielman et al., 2005). Notably, however, when subjects are aware of the presence and nature of the emotional stimuli these effects sometimes disappear (Niedenthal, 1990; Tamietto et al., 2006).

Psychophysiological changes that are associated with non-conscious perception of emotional stimuli include enhanced skin conductance (Esteves et al., 1994; Glascher and Adolphs, 2003) increased magnitude of eye blink (indicating startle reactions or avoidance) (Hamm et al., 2003), changes in stress hormone levels (van Honk et al., 1998), increased pupil dilation (Tamietto et al., 2009, 2015) and heart rate changes (Ruiz-Padial et al., 2005). These changes index arousal or the processing of affective valence, and their function is to prepare the organism for reacting to impeding and salient events. Notably, Amg lesions are associated with reduced eye blink to negative stimuli (Angrilli et al., 1996). Similarly, electromyography (EMG) studies have shown that masked or unseen emotional stimuli also trigger spontaneous facial reactions coherent with the emotional content of the stimuli (Dimberg et al., 2000; Tamietto and de Gelder, 2008b; Tamietto et al., 2009). This spontaneous tendency to synchronize our facial expressions with the emotional meaning of other individuals’ expressions is likely to play a part in social interactions (Frith, 2009).

A different source of evidence on the impact of stimulus processing without awareness comes from studies that used indirect manipulations. For example, indirect methods have been used to sample interference or integration between seen and unseen stimuli in patients with affective blindsight or during masking in healthy observers (de Gelder et al., 2001; Tamietto and de Gelder, 2008a; Bertini et al., 2013; Cecere et al., 2014). A typical example of indirect methods is the redundant target effect (RTE), in which one single stimulus is projected to the intact field or is presented simultaneously with another stimulus in the opposite blind field. Typically, reaction times (RTs) to the seen stimulus are faster during redundant stimulation than during single presentation to the intact field (Celeghin et al., 2015c). With such method, interactions between seen and unseen visual emotional stimuli, and also between (unseen) visual and (perceived) auditory stimuli, have been observed in such patients. For example, presenting an incongruent facial expression to the blind field biases the judgment of the emotional prosody of a sentence fragment (de Gelder et al., 2002, 2005), together with enhanced Amg activity during congruent conditions. These findings converge with the notion that emotion processing with and without stimulus awareness co-exist and interact in the intact brain, though they can be dissociated because of focal brain damage or experimental manipulation. Additional evidence on the motor influence of emotion perception is provided by transcranial magnetic stimulation (TMS) studies (Borgomaneri et al., 2015a,b). Although these studies did not manipulate directly visual awareness, they found extremely rapid sensory-motor modulation in response to fearful bodily expressions, supposedly underlying freezing mechanisms. As these effects are related to changes in the excitability of cortico-spinal downstream projections, but not in cortical excitatory mechanisms, the authors suggest that they are mediated by fast and automatic amygdala responses that rapidly modulate cortico-subcortical interactions before visual stimuli can be fully processed at a conscious level.

Can we experience consciously the bodily changes and emotional feelings determined by the exposure to an unseen and unperceived emotional stimulus? The classical view is that we become aware of such bodily responses when we can link them to the conscious representations of their external or internal determinants (e.g., an angry expression or a sudden noise, or our thoughts, respectively). In fact, some evidence indicates that we are unable to report a conscious feeling despite the fact that, at the same time, our behavior reveals the presence of an affective reaction triggered by the exposure to an external stimulus of which we are unaware. Despite this, however, it is conceivable that we can become aware of our physiological changes without a conscious representation of their external causes. This seems to be a common situation in clinical conditions such as alexithymia, pathological anxiety or depression. Also, one study on patients with affective blindsight has shown that the presentation of an unseen stimulus previously paired with an aversive event enhances eye-blink startle reflex, and this enhancement corresponded to the reported level of negative emotional feelings (Anders et al., 2004).

## Concluding Considerations

If emotional stimuli can be processed without awareness, activate the Amg, and still induce coherent responses, what role is left for consciousness in emotions? Some clues come from the observation that the responses observed when emotion processing is accompanied by awareness are often different from those induced by unconscious processing. Enhanced influence of non-consciously perceived emotional signals on physiological or expressive responses is in keeping with results showing that cortical activity and awareness can exert an inhibitory modulation over subcortical areas or automatic responses (Bush and Sejnowski, 1996; Panksepp, 2011). The fact that such inhibition is absent or less prominent during non-conscious perception of emotional stimuli could also explain the apparently paradoxical finding that subcortical activity can be enhanced during non-conscious compared to conscious perception of emotional stimuli in healthy subjects (Anderson et al., 2003; Williams et al., 2004b). Likewise, conscious perception of the eliciting stimulus can overrule subjective affective experience in response to an aversively conditioned stimulus, and the decoupling between conscious feelings and physiological changes correlates with increased activity in the ventro-lateral prefrontal cortex (Anders et al., 2009). These findings contradict the common assumption that emotional feelings merely reflect cortical readouts of peripheral and autonomic arousal. Therefore, the added value of awareness in emotion seems primarily that of integrating representations of the external and internal world in order to achieve context-dependent and higher-order decoupling and flexibility between sensory input and behavioral output. Consciousness also allows control and planning, as well as anticipation of desirable or functional responses.

From the opposite vantage point, emotions seem to play a prominent role in the generation and development of state consciousness, which refers to the different degrees of vigilance, such as wakefulness, alertness, drowsiness, or coma that apply to the whole organism. Our homeostatic regulation depends indeed by the continuous mapping of bodily states and integration of interoceptive information. These homeostatic processes contribute to generate the sense of invariance that accompanies contingent subjective experience, and thus instantiate a neurobiological mechanism for the invariance of the sense of self and the continuity of our first-person experience of the world (Damasio, 1999; Tsuchiya and Adolphs, 2007; Park and Tallon-Baudry, 2014). Neurophysiological responses induced by emotional signals, even when they are unseen, alter homeostatic balance and overlap with changes affecting the general level of state consciousness (Damasio, 1999; Zeman, 2001; Damasio and Carvalho, 2013). It is indeed noteworthy that the bodily responses triggered by emotions are controlled by neural structures in the brainstem that also control the level of consciousness. Accordingly, several scholars consider raw emotional feelings as the precursors or basic forms of consciousness, and have rooted it in subcortical processes rather than (only) in full-blown subjective cognitions implemented in higher-order cortical structure (Panksepp, 2005; Panksepp, 2011; Damasio and Carvalho, 2013; Damasio et al., 2013; LeDoux, 2015). In keeping with this perspective, children with total congenital absence of the cerebral cortex can nevertheless exhibit appropriate affective responses and feelings can be even strengthened (Shewmon et al., 1999). Moreover, direct electrical brain stimulation in subcortical and brainstem structures that evoke observable behavioral and physiological reactions associated with reward and punishment in animals, also induce conscious affective feelings when stimulated in humans (Panksepp, 2005; Panksepp, 2011). Thus, also when we are not aware of the external determinants of an emotional response, because the triggering signal does not become a content of our conscious visual experience, the cascade of physiological reactions it generates in the organism contributes to modulate our state of vigilance and behavior, which are constitutive components of state consciousness.

## Author Contributions

MD, AC, AB, and MT wrote the manuscript. MT revised the final version of the manuscript. All authors agreed on the final version of the manuscript

## Conflict of Interest Statement

The authors declare that the research was conducted in the absence of any commercial or financial relationships that could be construed as a potential conflict of interest.
