# Supplementary material for: Amygdala Response to Emotional Stimuli without Awareness: Facts and Interpretations
Source: Front Psychol. 2017 Jan 10;7:2029. doi: 10.3389/fpsyg.2016.02029 (PMC5222876; doi:10.3389/fpsyg.2016.02029)
Supplement: Supplementary file 1 [file Table_1.DOCX]

**Table 1**

Overview of the studies reporting amygdala response during sensory and attentional unawareness for emotional stimuli

| **First Author** | **Year** | **Journal** | **Type of Unawareness** | **Sample** | **Measures** | **Paradigm** | **Stimuli** | **Main Amygdala Effects During Unawareness** |
| --- | --- | --- | --- | --- | --- | --- | --- | --- |
| Anderson | 2001 | Nature | Attentional | Healthy + Patients with Amg lesion | Behavioural | Attentional blink | words | Negative > Neutral in controls  Negative = Neutral in Amg lesion patient |
| Anderson | 2003 | J Neurosci | Attentional | Healthy | fMRI | Focused attention with superimposed coloured images | faces  (fearful /angry/neutral) + places | R: fearful > neutral; disgust > neutral |
| Bishop | 2007 | Cer Cortex | Attentional | Healthy + Patients with state anxiety | fMRI | Focused attention with superimposed images | faces (fearful/neutral) + letters | R: fearful > neutral in low perceptual load; L: fearful > neutral in low perceptual load for high state anxiety group |
| Bishop | 2004 | J Neurosci | Attentional | Healthy + Patients with state anxiety | fMRI | Dual-Task with focused attention and same-different discrimination | faces (fearful/neutral) + houses | L: fearful > neutral in high state anxiety group |
| Carlson | 2009 | Neuropsychologia | Sensory | Healthy | fMRI | dot-probe task with backward masking | faces (fearful/neutral) | L: fearful > neutral |
| Carlson | 2010 | Psychiatry Research: Neuroimaging | Sensory | Healthy | fMRI | backward masking | faces (fearful/neutral) | L: fearful > neutral |
| Carlsson | 2004 | Emotion | Sensory | Phobic Patients | PET | backward masking | spiders + snakes + faces (neutral) | L: phobic feared or non-phobic fearful stimuli > neutral faces |
| Critchley | 2000 | Hum Brain Map | Attentional | Healthy | fMRI | gender discrimination | faces (happy/angry/neutral) | Bil: happy + angry > neutral |
| Dannlowski | 2007 | J Psychiatry Neurosci | Sensory | Patients with MDD | fMRI | backward masking + affective priming | faces (angry/happy/sad/neutral) | Bil: sad > no face; angry > no face; R: happy > no face; Bil: sad > neutral; angry > neutral correlated with more negative bias score |
| de Gelder  (Exp. 1) | 2005 | Proc Natl Acad Sci USA | Sensory | Blindsight Patients | fMRI | RTE | faces + chimeric faces (fearful/happy) | L: bilateral congruent fearful faces > bilateral congruent happy faces R: bilateral congruent fearful hemifaces > bilateral congruent happy hemifaces |
| de Gelder  (Exp. 2) | 2005 | Proc Natl Acad Sci USA | Sensory | Blindsight Patients | fMRI | blind field presentation | faces (fearful/happy) + IAPS | R: fearful > happy faces R: fearful/happy faces > fearful/happy IAPS images |
| de Gelder  (Exp. 3) | 2005 | Proc Natl Acad Sci USA | Sensory | Blindsight Patients | fMRI | cross-modal RTE | faces (fearful/happy) + IAPS + voice fragments | R: fear face/voice congruence > incongruence; R: face/voice congruence > scene/voice congruence |
| Dickie | 2008 | Psychiatry Res Neuroimaging | Attentional | Healthy + Subjects with trait anxiety | fMRI + Anxiety scores | Focused attention with superimposed images | faces (fearful/neutral) + indoor and outdoor scenes | R: fearful unattented > fearful attended in female with high anxiety |
| Duan | 2010 | Neuroimage | Sensory | Healthy + Alexithymic Subjects | fMRI + Alexithymia scales | backward masking | superimposed faces (happy/neutral) | R: surprise > neutral; surprise > happy; L: happy > neutral |
| Etkin | 2004 | Neuron | Sensory | Healthy | fMRI + Anxiety scores | backward masking | faces (fearful/neutral) | R baso-lateral: fearful > neutral + positive correlation anxiety |
| Ewbank | 2009 | NeuroImage | Attentional | Healthy | fMRI + Anxiety scores | Dual-Task with focused attention and same-different discrimination | faces (fearful/angry/neutral) + houses | L: fear > neutral; fear > anger in higher anxiety participants |
| Faivre | 2012 | Neuropsychologia | Attentional | Healthy | fMRI | affective priming | static and dynamic faces (happy/neutral) | Bil: static happy > neutral; increased amy FFA connectivity |
| Fan | 2011 | NeuroImage | Sensory | Healthy | fMRI | affective priming | faces (fearful/neutral) + oval shapes | R: fearful or neutral primes > oval primes |
| Fang | 2016 | Front Hum Neurosci | Sensory | Healthy | fMRI | backward masking | negative and neutral non human animals + negative and neutral objects | Bil: negative pictures > neutral; L: negative animals > negative objects; Bil: category by emotion interaction |
| Garrido | 2012 | Curr Biol | Attentional | Healthy | MEG | focused attention with gender discrimination | faces (fearful/happy/neutral) + sounds | Amy response better explained by a rapid subcortical and slower cortical pathway, rather than by a cortical pathway only |
| Gavert | 2014 | NeuroImage | Attentional | Healthy | MEG | focused attention with gender discrimination | faces (fearful/happy/neutral) + sounds | Amy response better explained by a rapid subcortical and slower cortical pathway, rather than by a cortical pathway only |
| Glascher | 2003 | J Neurosci | Sensory | Healthy + Patients with Temporal Damaged | SCR/EOG | backward masking | IAPS | RTD: impaired SCR; LTD: impaired correlation of SCR with the rated arousal of the stimuli |
| Harmer | 2006 | Biol Psychiatry | Sensory | Healthy + Patients with SSRI | fMRI | backward masking | faces (fearful/happy/neutral) | R: fear placebo > drug |
| Juruena | 2010 | J Int Neuropsychol Soc | Sensory | Healthy | fMRI | backward masking | faces (happy/sad/neutral) | Bil: happy > sad; L: happy > neutral |
| Killgore | 2004 | NeuroImage | Sensory | Healthy | fMRI | backward masking | faces (happy/sad/neutral) | Bil: happy; R: happy > sad; L: happy + sad |
| Killgore | 2007 | Soc Neurosci | Sensory | Healthy (children/adolescents/adults) | fMRI | backward masking | faces (happy/sad/neutral) | R: sad; R: sad in adolescents > sad in adults |
| Killgore | 2014 | Depress Anxiety | Sensory | Healthy+ Patients with PTSD, PD, or SP | fMRI | backward masking | faces (happy/fearful/neutral) | fear vs. neutral  L: patients > control, PTSD > control; happy vs. neutral  L: patients > control, PTSD > control; fear vs happy  L: control > SP |
| Kim | 2010 | Soc Cogn Affect Neurosci | Sensory | Healthy | fMRI | backward masking | faces (happy/fearful/neutral) | R: fear > happy |
| Liddell | 2005 | NeuroImage | Sensory | Healthy | fMRI | backward masking | faces (fearful/neutral) | Bil: fear > neutral |
| Lou | 2010 | J Neurosci | Attentional | Healthy | MEG | Dual-Task with focused attention and same-different discrimination | faces (fearful/neutral) + lines | early amy activity to fear unaffected by attentional load |
| McCroy | 2013 | Br J Psychiatry | Sensory | Healthy + Maltreated Children | fMRI | backward masking | faces (angry/happy/neutral) | R: maltreated vs. non-maltreated fear > neutral; happy > neutral |
| Morris | 2001 | NeuroImage | Sensory | Healthy | fMRI | backward masking + conditioning | faces (angry/neutral) + aversive noise | L: angry masked > unmasked |
| Morris | 1998 | Nature | Sensory | Healthy | PET | backward masking + conditioning | faces (angry/neutral) + aversive noise | R: angry conditioned > non-conditioned |
| Morris | 2001 | Brain | Sensory | Blindsight Patients | fMRI | blind field presentation + conditioning | faces (fearful/happy/angry) + aversive noise | Bil: fear > happy; Bil: angry conditioned > non-conditioned |
| Morris | 1999 | Proc Natl Acad Sci USA | Sensory | Healthy | PET | backward masking + conditioning | faces (angry/neutral) + aversive noise | R: conditioned > non-conditioned |
| Nomura | 2004 | NeuroImage | Sensory | Healthy | fMRI | backward masking + affective priming | faces (angry/neutral) | R: anger primes > baseline, anger primes > neutral primes |
| Novak | 2015 | Neuropsychologia | Sensory | Healthy | fMRI | face and odour morphing | faces (fearful/neutral) + odor (negative/neutral) | R: face and odour negative > neutral; R: congruent face/odours > incongruent |
| Pasley | 2004 | Neuron | Attentional | Healthy | fMRI | binocular rivalry | houses, chairs, faces (fearful) | L: suppresed face > suppressed chairs |
| Pegna | 2005 | Nat Neurosci | Sensory | Blindsight Patients | fMRI | blind field presentation | faces (fearful/anger/happy/neutral) | R: fear/anger/happy > neutral |
| Pichon | 2012 | NeuroImage | Sensory | Healthy | fMRI | backward masking + priming | faces (fearful/neutral) + houses + words | R: emotional priming > neutral priming |
| Sagaspe | 2011 | NeuroImage | Attentional | Healthy | fMRI | stop-signal task | faces (fearful/neutral) | R: fearful > neutral |
| Sato | 2010 | Brain Res | Sensory | Healthy | fMRI | masking | dynamic faces (fearful/happy/neutral) | L: forward > backward presentation for both fearful and happy expressions. |
| Silvert | 2007 | NeuroImage | Attentional | Healthy | fMRI | Dual-Task with focused attention and same-different discrimination | faces (fearful/neutral) + houses | R: unattended fearful > neutral faces in the low-load task |
| Straube | 2010 | Hum Brain Mapp | Sensory | Healthy | fMRI | backward masking | eyes (fearful/happy) | L: fearful vs. happy |
| Suslow | 2013 | Psychiatry Res Neuroimaging | Sensory | Healthy | fMRI | backward masking | faces (sad/ happy/neutral) | Bil: sad > neutral |
| Tamietto | 2015 | Cortex | Attentional | Patients with Hemispatial Neglect | fMRI + pupil dilation | visual extinction | bodies (neutral/fearful) | L: extinguished fearful > unilateral RVF neutral |
| Troiani | 2013 | Front Hum Neurosci | Sensory | Healthy | fMRI | continuous flash suppression | faces (fearful) + houses | R: fearful faces or houses > baseline |
| Troiani | 2014 | Soc Cogn Affect Neurosci | Sensory | Healthy | fMRI | continuous flash suppression | faces (fearful) + houses | L: fearful faces > houses |
| Van den Stock | 2011 | Proc Natl Acad Sci USA | Sensory | Blindsight Patients | fMRI | blind field presentation | body movies (angry/neutral) | Bil: anger > neutral |
| Victor | 2012 | Plos ONE | Sensory | Patients with MDD | fMRI | backward masking | faces (sad/happy/neutral) | Bil: sad > happy faces in MDD vs. healthy controls |
| Victor | 2010 | Archf Gen Psychiatry | Sensory | Patients with MDD | fMRI | backward masking | faces (sad/happy/neutral) | Bil: sad > happy faces in MDD vs. healthy controls  Bil: happy > neutral faces in healthy controls vs. MDD |
| Viding | 2012 | Am J Psychiatry | Sensory | Subjects with Conduct problems | fMRI | backward masking | faces (fearful/neutral) | R: fear > calm, in conduct problems/low callous-unemotional traits |
| Vuilleumier | 2001 | Neuron | Attentional | Healthy | fMRI | Dual-Task with focused attention and same-different discrimination | faces (fearful/neutral) + houses | Bil: fearful > neutral faces |
| Vuilleumier | 2002 | Neuropsychologia | Attentional | Patients with Hemispatial Neglect | fMRI | visual extinction | faces (fearful/neutral) + houses | L: extinguished fearful faces > unilateral RVF house |
| Whalen | 2004 | Science | Sensory | Healthy | fMRI | backward masking | eyes (fearful/happy) | L: fearful > happy |
| Whalen | 1998 | J Neurosci | Sensory | Healthy | fMRI | backward masking | faces (fearful/happy) | Bil: fearful > happy faces |
| Williams | 2005 | NeuroImage | Attentional | Healthy | fMRI | Dual-Task with focused attention and same-different discrimination on superimposed images | faces (fearful/happy) + houses | R: unattended fearful > attended fearful |
| Williams | 2006 | Hum Brain Mapp | Sensory | Healthy | fMRI + SCR | backward masking | faces( fearful/neutral) | Bil: fearful > neutral |
| Williams | 2006 | J Neurosci | Sensory | Healthy | fMRI + SCR | backward masking | faces (fearful/neutral) | Bil: fearful > neutral |
| Williams | 2004 | J Neurosci | Sensory | Healthy | fMRI | binocular rivalry | faces (fearful/happy/ neutral) + houses | Bil: fearful > neutral faces;  R: happy > neutral faces |
| Yang | 2012 | Brain Cogn | Sensory | Healthy | fMRI | backward masking | faces (fearful/neutral) | R: fearful > neutral |

*Abbreviations:* Bil = bilateral; EOG = electro-oculogram; fMRI = functional magnetic resonance imaging; IAPS = international affective pictures stimuli; L = left amygdala; LVF = left visual field; MDD = major depressive disorder; MEG = magnetic encephalography; PD = panic disorder; PTSD = posttraumatic stress disorder; R = right amygdala; RTD, LTD, BLD = right, left, bilateral temporal damage; RTE = redundant target effect; RVF= right visual field; SCR = skin conductance responses; SP = specific animal phobia; SSRI = selective serotonin reuptake inhibitor;
